# Tunable Infrared Optical Switch Based on Vanadium Dioxide

**DOI:** 10.3390/nano11112988

**Published:** 2021-11-06

**Authors:** Qi Wang, Shijie Zhang, Chen Wang, Rui Li, Tianhan Cai, Dawei Zhang

**Affiliations:** Shanghai Key Laboratory of Modern Optics System, Engineering Research Center of Optical Instrument and System, Ministry of Education and Shanghai Key Laboratory of Modern Optical System, School of Optical-Electrical and Computer Engineering, University of Shanghai for Science and Technology, 516 Jungong Rd., Shanghai 200093, China; 202310334@st.usst.edu.cn (S.Z.); 183740566@st.usst.edu.cn (C.W.); 192380338@st.usst.edu.cn (R.L.); 1935031109@st.usst.edu.cn (T.C.); dwzhang@usst.edu.cn (D.Z.)

**Keywords:** vanadium dioxide, localized surface plasmon resonance, optical switch, mid-infrared

## Abstract

A tunable infrared optical switch based on a plasmonic structure consisting of aluminum nanoarrays with a thin film of vanadium dioxide is proposed. This optical switch can realize arbitrary wavelength-selective optical switching in the mid-infrared region by altering the radii of the aluminum nanoarrays. Furthermore, since vanadium dioxide transforms from its low-temperature insulator phase to a high-temperature metallic phase when heated or applied voltage, the optical switch can achieve two-way switching of its “ON” and “OFF” modes. Finite-difference time-domain software is used to simulate the performance of the proposed infrared optical switch. Simulation results show that the switch offers excellent optical performances, that the modulation depth can reach up to 99.4%, and that the extinction ratio exceeds −22.16 dB. In addition, the phase transition time of vanadium dioxide is on the femtosecond scale, which means that this optical switch based on a vanadium dioxide thin film can be used for ultrafast switching.

## 1. Introduction

With the continuing development of optical communication technologies and internet protocol (IP) networks, all-optical IP networks have become the core research of the optical communications industry. Optical switches are used to realize the switching functions of the optical network and have been widely applied in the field of electronics. Optical switch is one of the most important components in the optical communication network. High-speed optical switches and modulators with various functions have been developed in recent years [1,2]. Meanwhile, nanomaterials composed of tiny micro-units of molecular or even atomic sizes have been developed. Compared with conventional materials composed of the same elements, nanomaterials offer unique chemical and physical properties, including their electrical, optical, and magnetic properties; thus, these materials have been widely used in numerous fields [3]. Nanomaterials have enabled the application of surface plasmon resonance (SPR), guided-mode resonance, magnetic polaritons (MPs), Fano resonance, and other effects in the development of optical components with various functions, including super lenses [4], filters [5], absorbers [6], and modulators [7]. An optical switch is an optical element that can regulate optical signals in “ON” and “OFF” modes. Switching between these two modes can be performed through heating or controlling the applied electric field.

Phase change materials (PCMs) are widely used in the field of the thermally controlled optical switches due to the unique optical and infrared properties that they offer in the different input phases. The input phase of PCM can be changed by controlling the temperature and the electric field applied to the material. Vanadium dioxide (VO_2_) is a classic PCM that possesses metal-insulator transition characteristics [8]. The phase transition temperature of VO_2_ is 68 °C, which is defined as the critical temperature, T_C_. Below this temperature, VO_2_ is in an insulating monoclinic phase; otherwise, the material is in a metallic rutile phase. Meanwhile, VO_2_ has ultra-high phase transition speed and near-room-temperature phase transition, and VO_2_ thin-film deposition technology is also viable. These are very attractive qualities for use in the development of optical switches. The transition time from the metallic state to the semiconductor state in a VO_2_ thin film is approximately 150 fs [9,10], which is an order of magnitude faster than the switching times of mechanical switches and acousto-optic switches. Moreover, the phase transition temperature of VO_2_ is 68 °C, which is closer to room temperature than the phase transition temperatures of other PCMs, such as Ge_2_Sb_2_Te_5_ (GST, 160 °C), which means that VO_2_ can lower the temperature requirements and broaden the application fields of optical switches. Besides, the phase transition temperature of the VO_2_ thin films can be varied by doping the films with noble metals [11], which allows the temperature to change according to the specific application.

When selecting materials for plasmonic structures, it is necessary to consider both the costs and the durability of the materials. Metallic gold (Au) and silver (Ag) are the two most commonly used materials for plasmonic structures in the range of visible and infrared wavelengths [12,13]. Au is a chemically inert metal and therefore offers high durability, whereas metallic Ag reacts with the oxygen and sulfur content in the atmosphere, which causes the degradation of its plasmonic characteristics over time. However, these two metals are relatively expensive, which is not conducive to device production. In addition, Au and Ag have inherent absorptivity properties that will limit the adjustability of the localized surface plasmon resonance (LSPR) [14,15]. For example, the inherent absorption wavelengths of Au and Ag are 500 and 400 nm, respectively. As an alternative, aluminum (Al) is a low-cost and resource-rich material when compared with the noble metals described above, and the Al surface naturally grows a 3–5 nm-thick termination oxide layer that can protect it from oxidation and improve its durability. Therefore, Al is also the most suitable choice for plasmonic devices.

Recently, optical switches based on VO_2_ have been extensively investigated. For example, Thomas et al. [16] presented a novel optical switch that consists of an Au nanowire-VO_2_ spacer-Au film. However, the optimal switching wavelength can be arbitrarily selected in a wide spectral range by changing the structural parameters of the proposed optical switch. The modulation depth (MD) of the optical switch is less than 75%, while optical switches that have a larger MD and extinction ratio (ER) are required in optical communications (definitions of MD and ER are described in the following context).

In this paper, we present a novel infrared optical switch and propose a modulator that can display both optical switching and modulation characteristics in the mid-infrared band and also offers ultra-fast switching. The proposed optical switch uses the phase change characteristics of VO_2_, which has high reflectivity in its metallic phase and almost zero reflectivity otherwise. When VO_2_ is in its insulator phase, we couple the incident light waves to the LSPR of the Al nano-disk, which means that the reflectivity of the optical switch is almost zero when it is in the insulator phase. The optical switch in the “ON” mode has a reflectivity of approximately 0.8, but when the optical switch is in the “OFF” mode, the reflectivity is approximately 0.002. These phenomena prove that the proposed optical switch offers our intended results, which have larger MD and ER. In order to demonstrate that, by changing the structural parameters, the wavelength where the maximum differential reflectance exists can be selected over a wide spectral range. The numerical simulations are employed to explore the influence of various geometric parameters of the Al nano-disk radius, *R,* and the period, *Λ,* on the resonance wavelength of the optical switch. Additionally, the angular insensitivity of the structure is investigated for the TM and TE polarizations, and the resonant frequency of the optical switch will not vary with changes in the incident angle within a specified range. Due to the excellent optical switching and modulation performances, the device has a potential application in optical communication networks, optical modulators, and so on.

## 2. Structure and Design

**Figure 1:**
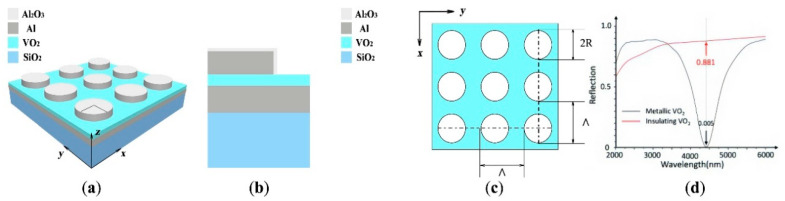
(**a**,**b**) Structure of the proposed wavelength-selective optical switch. The thicknesses of the VO_2_ film, the Al layer, and the SiO_2_ film are 50, 180, and 300 nm, respectively. (**c**) Top view of the switch structure. The radius of the Al nano-disk (surrounded by Al_2_O_3_ film with a thickness of 3 nm) is *R* = 300 nm, its height *d_Al Nanodisk_* = 220 nm, and the period of the structure *Λ* = 800 nm. (**d**) Spectral reflectance characteristics of the optical switch for TM polarized light at oblique incidence before and after the VO_2_ phase change. Here, the VO_2_ phase is changed through a temperature change to adjust the ON and OFF modes of the optical switch.

The wavelength-selective optical switch proposed in this work adopts a periodic four-layer structure, as shown in Figure 1a,b. From the bottom to the top, the structure is composed of a SiO_2_ substrate, an Al metal layer, a VO_2_ film layer, and an Al nanoarray (surrounded by Al_2_O_3_ film with a thickness of 3 nm, due to the fact that the Al surface naturally grows a 3–5 nm-thick termination oxide layer that can protect it from oxidation and improve its durability). It should be noted that the thickness of the VO_2_ film in the proposed structure is less than 50 nm, in order to couple light directly into VO_2_ between the Al nanoarray and the underlying Al film [16]. The entire structure is supported by a SiO_2_ substrate. The corresponding thicknesses of these four layers are 300, 180, 50, and 220 nm, respectively. A 180 nm thickness of the Al layer is selected to fully block the incident light without transmission. Figure 1c shows the Al nanoarray layer composed of Al nano-disks with *R* = 300 nm, while the period, Λ, on both the *x*-axis and the *y*-axis is 800 nm.

In our simulation, commercial software Lumerical for the finite-difference time-domain (FDTD) method was used to analyze the reflectance performance of the switch structure. The Al nanoarray structure with a thin VO_2_ layer was simulated to determine its reflectance as a function of the wavelength. A plane wave with transverse magnetic (TM) polarization and a wavelength, λ, propagate along the +*z* direction onto the optical switch at an incident angle *θ*. The reflectance spectra of the structure were monitored using a power monitor placed above the light source. Since the optical switch is periodic along the *x-* and *y*-axes, periodic boundary conditions are set on both the *x-* and *y*-axes, while a perfectly matched layer (PML) is selected on the *z*-axis. The space in the *x*, *y*, and *z* directions is divided using a mesh interval equal to Δ*x* = Δ*y* = Δ*z* = 2 nm, and the numerical convergence criterion is less than 10^−6^ to minimize any numerical errors.

The complex refractive indices of Al, SiO_2_, and Al_2_O_3_ are used from the data of Palik [17]. The functional relationship between the optical constants of the VO_2_ film, ε(ω), can be obtained through the use of the classical dispersion model based on the Lorentz and Drude oscillators, which are expressed by Equation (1) [18]:

(1) $$\varepsilon (\omega )=\varepsilon_{\infty }-\frac{{\omega_{n}}^{2}}{\omega^{2}+i\omega_{c}\omega }+\overset{n}{\underset{i=1}{\Sigma }}\frac{s_{i}}{1-\omega^{2}/{\omega_{i}}^{2}-i\Gamma_{i}\omega /\omega_{i}}$$

The constant $\varepsilon_{\infty }$ is a dielectric constant from high frequency, $\omega_{n}={\left(4\pi n_{c}e^{2}/m^{*}\right)}^{1/2}$ is the carrier density parameter related to the plasma frequency, $\omega_{c}=e/\mu_{\mathrm{opt}}m^{*}$ is the collision frequency, where $n_{c}$ is the number of conduction electrons and $m^{*}$ denotes the so-called optical mass of the electrons defined by Cohen, and $s_{i}$, $\omega_{i}$*,* and $\Gamma_{i}$ are the strength, frequency, and line-width of the *i*-th oscillator, respectively.

Figure 1d depicts the reflectance spectra of the optical switch for the two different phases of the VO_2_ film, when the structure parameters are selected at *d*_SiO_2__ = 300 nm, *d*_Al_ = 180 nm, *d*_VO_2__ = 50 nm, *d*_Al Nanodisk_ = 220 nm, *R* = 300 nm, and *Λ* = 800 nm. The incident wave is a transverse magnetic (TM) polarized wave with the incident angle of 45°. When VO_2_ is in the semiconductor phase (where the temperature is lower than the critical temperature, T_C_), a reflection dip occurs at the wavelength of 4410 nm, and the reflectivity is 0.005. However, when VO_2_ is transformed into its metallic phase (where the temperature is higher than the critical temperature, T_C_), the reflectivity reaches as high as 0.881 at the same wavelength. Therefore, the “ON” and “OFF” modes of the optical switch can be controlled by simply adjusting the temperature of the VO_2_ film.

**Figure 2:**
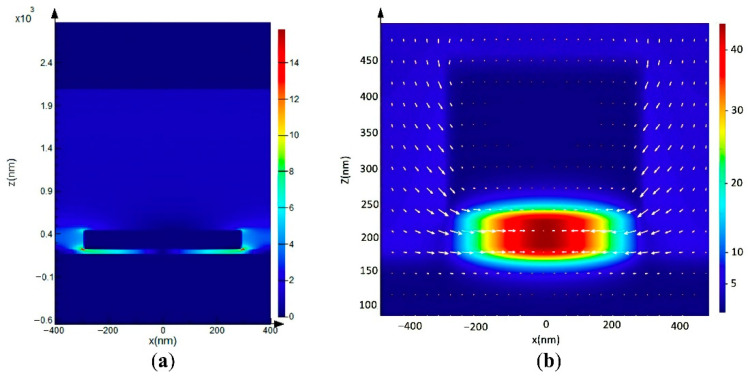
The electric field intensity distribution and the magnetic field intensity distribution at the resonant wavelength of the proposed optical switch structure: (**a**) when the VO_2_ film is in the insulator phase, the electric field distribution at the cross-section is given along the *z*-axis, and (**b**) the magnetic field distribution at the resonant wavelength

In order to study the physical mechanisms of the wavelength-selective optical switch, we set the optical switch parameters as follows: *d*_SiO_2__ = 300 nm, *d*_Al_ = 180 nm, *d*_VO_2__ = 50 nm, *d*_AlNanodisk_ = 220 nm, *R* = 300 nm, and *Λ* = 800 nm, and the angle of incidence is 45° under TM mode. The electromagnetic field distributions of the two VO_2_ phases (i.e., the insulator phase and the metal phase) at the resonant wavelength were investigated as shown in Figure 2. When the VO_2_ film is in its insulator phase, the electric field distribution at the resonance wavelength is as shown in Figure 2a, where it can be seen clearly that the electric field is limited at the boundary of the Al nano-disk. The electric field distribution indicates the appearance of LSPR, which is a phenomenon where the frequency of the photons of incident light on a metal surface matches with the natural oscillation frequency of the electrons on that surface, and the incident light induces collective oscillation behavior in the charges at the metal–insulator interface [15]. In particular, when the size of the aluminum nanoparticles is much smaller than the incident light wavelength and the surface-free electrons induced by the incident photons and the Al nanoarrays oscillate at the same frequency, a resonance peak will be generated to enhance the collective resonance of the surface electrons. Therefore, the aluminum nanoparticles can absorb both visible and infrared light within a specific range [19], and show a strong characteristic absorption peak within the visible and infrared absorption spectra at the macro-level. Due to the LSPR effect, the aluminum nanoarray absorbs photons, which causes the local electromagnetic field at the nano-disk boundary to be enhanced.

Figure 2b depicts the magnetic field distribution at the resonant wavelength. When the incident photons and the free electrons on the surfaces of the aluminum nanoarrays oscillate at the same frequency, a magnetic resonance effect will be produced, and the resulting magnetic coupling will produce a magnetic moment that interacts with the incident optical magnetic field in the VO_2_ film layer. Therefore, when VO_2_ is in its insulator phase, the incident light waves are limited in the optical switch, which means that the reflectivity of the optical switch is then close to zero.

This mode coupling behavior can be controlled via the size and the height of the Al nano-disks [20,21,22,23]. By changing these variables, it is possible to control the resonance wavelength selection process.

## 3. Results and Discussion

**Figure 3:**
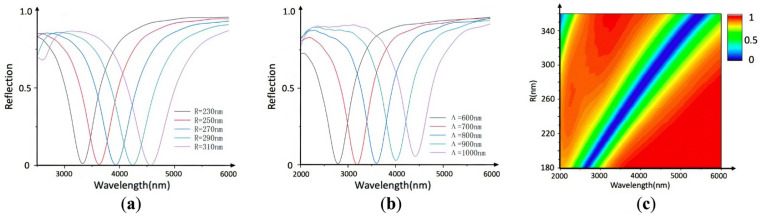
VO_2_ film in its insulator phase. (**a**) Change in the reflectance spectrum versus different values of radius *R*. (**b**) Change in the reflectance spectrum versus the wavelength of the optical switch with disk duty ratio *f* = 0.3, where the reflectance is calculated for Al nano-disks with different values of the period *Λ* (where *Λ* = 600, 700, 800, 900, and 1000 nm). *d*_VO_2__ = 50 nm. (**c**) Reflection spectra of the optical switches with different radii.

To explore the influence of the Al nano-disk radius on the resonant wavelength of the optical switch, we selected the five *R* values of 230, 250, 270, 290, and 310 nm in Figure 3a, while keeping other parameters constant. It depicts a redshift in the resonance wavelength with increasing nano-disk radius in the mid-infrared band when the VO_2_ film is maintained at the same temperature (insulator phase).

Additionally, to evaluate the influence of the period of the Al nano-disk on the resonant wavelength of the optical switch, we vary the period of the Al nanoarray, while maintaining the duty cycle (the duty cycle is defined as the ratio of the radius of the Al nano-disk, *R,* to the period, *Λ*, which is called the disk duty ratio *f* = 0.3 in this work). Five period values of 600, 700, 800, 900, and 1000 nm are selected in steps of 100 nm, and the results are shown in Figure 3b. It can be seen from Figure 3c that when VO_2_ is maintained at the same temperature (i.e., in the insulator phase), the resonance wavelength in the mid-infrared band is red-shifted with the increasing nano-disk radius.

**Figure 4:**
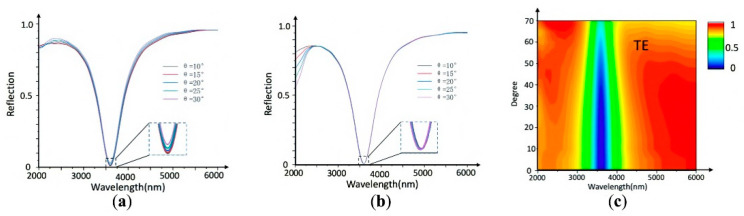
Spectral reflectance curves of the optical switch at different incident angles for the (**a**) TE polarization and (**b**) TM polarization. The incident angle steps in 5°, from 10° to 30°. (**c**) Reflectivity of the optical switch at different incident angles for TE polarized light.

Ideally, the reflection properties of a perfect optical switch should be able to tolerate a wide range of angles of incidence and also exhibit polarization-independent features. Figure 4a,b present the reflection characteristics for the TE and TM polarizations respectively, revealing their dependences on the angle of incidence. We can see that the optical switch has good angle-insensitive characteristics for both the TE and TM polarizations. When the incident angle increases, the reflectivity also shows a slight increase. However, in contrast to the TE polarization, the TM polarization is more insensitive to the incident angle and the reflectivity almost does not increase at all. Figure 4c clearly shows that the influence of the TE-polarized light incident on the light switch at the resonant frequency is small, with a wide angular range from 0° to 70°. Excellent angular independence and polarization independence provide convenient conditions for the application of the optical switch in optical communication technology.

To quantify the efficacy of the resulting optical switch, we define the wavelength-dependent MD and ER [24] as:

(2) $$\mathrm{MD}(\lambda )=\frac{R_{\mathrm{on}}(\lambda )-R_{\mathrm{off}}(\lambda )}{R_{\mathrm{on}}(\lambda )},$$

(3) $$\mathrm{ER}(\lambda )=-10\mathrm{lg}\frac{R_{\mathrm{on}}(\lambda )}{R_{\mathrm{off}}(\lambda )},$$

Here, *R*_on_ (*λ*) and *R*_off_ (*λ*) represent the maximum and minimum reflectivity, respectively. Obviously, when the ratio of *R*_on_ (*λ*) and *R*_off_ (*λ*) is higher, the reflection difference is more obvious, and the performance of the optical switch is better. Table 1 shows the ER at the resonance wavelength when the radius of the Al nano-disk changes from 180 to 360 nm. The FDTD simulation results indicate that the MD of the optical switch designed in this paper reaches 99.4% and the ER is −22.16 dB, which has significant potential for the actual optical switch application.

There have been many international studies on optical switches and modulators based on PCMs [16,25,26], liquid crystal (LC) [27], graphene [28], and so on [29,30,31]. Table 2 shows a comparison of the MD and ER of optical switches and modulators reported by several groups. These performance parameters in the table exhibit the excellent modulation characteristics of the proposed structure compared to some previous studies. Moreover, the LSPR character of our optical switch enables a straightforward geometrical wavelength tunability to excite efficiently switching characteristics for a wide range of operating wavelengths, which has been confirmed in this work by changing the geometric parameters of the Al nano-disk radius, *R,* and the period, *Λ*.

**Figure 5:**
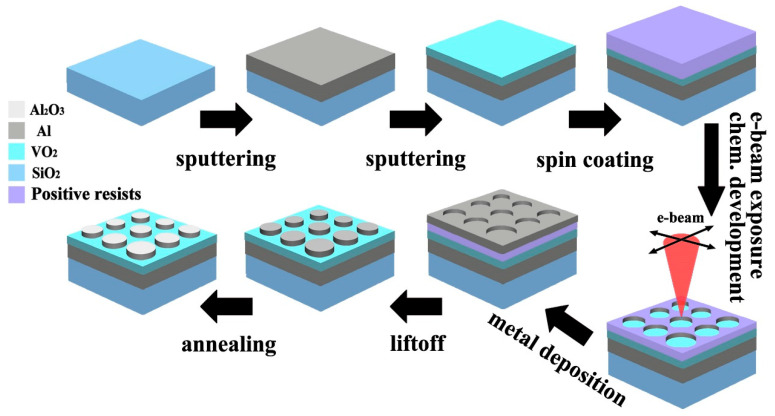
Fabrication flow for realization of the proposed optical switch.

Although we only study from the perspective of simulation, the experimental feasibility is also analyzed. Figure 5 shows the preparation process of the optical switch based on the magnetron sputtering technique and the electron beam lithography. Firstly, the Al and the VO_2_ films can be fabricated by sputtering the related materials on the SiO_2_ substrates in turn. Secondly, a layer of photoresist was coated on the films and exposed, developed by the electron beam lithography to form the periodic nano-disks, and the Al nano-disks can be fabricated by successively sputtering Al on the photoresist strip array. Then, the remaining photoresist was removed by the lift-off method. In addition, the protective cap layer of Al_2_O_3_ was obtained through the oxidation of the Al nano-disks array.

## 4. Conclusions

In this paper, we have designed an infrared optical switch based on an array of aluminum nano-disks on top of a VO_2_ thin-film layer. When the temperature is below 68 °C, the optical switch has low reflectivity at the switching wavelength because of the excitation of LSPR, and the switch is in the “OFF” mode. In contrast, when the temperature of the VO_2_ film exceeds 68 °C, the optical switch has higher reflectivity, and the switch is in the “ON” mode. The proposed device plays a key role in the optical network and electronics for high-capacity communications, thermo-optic modulators, and optical cross-connect. In addition, for the TM and TE polarizations, the resonant frequency of the optical switch will not vary with changes in the incident angle within a certain range, and the operating wavelength of the optical switch can be adjusted by varying the geometric parameters of the Al nano-disks. This results in angular-independent and spectral-selective optical switching properties, ideally suited for applications in the optical communications industry, industrial process monitoring, and so on. Simulations performed with FDTD Solutions software showed that the structure of the optical switch offers an excellent optical switching performance with an outstanding modulation depth of 99.4% and an extinction ratio of −22.16 dB. This study can serve as a valuable reference for optical switching technology development.

## Figures and Tables

**Table 1:** Resonance wavelength and extinction ratio of optical switches with different radii.

Radius (nm)	Wavelength (nm)	ER (dB)
180	2638	−12.40
200	2910	−14.68
220	3195	−17.01
240	3486	−19.03
260	3785	−20.63
280	4096	−21.54
300	4410	−22.16
320	4745	−22.04
340	5101	−21.41
360	5494	−20.18

**Table 2:** Comparison of modulation performances between this work and previous works.

Reference	Modulation Material	MD	ER (dB)
Gholipour et al. [25]	GST	60.0%	−3.98
Bian et al. [27]	LC	98.3%	−17.70
Zeng et al. [28]	graphene	~90.0%	-
Xie et al. [29]	Indium tin oxide	97.8%	−16.65
Thomas et al. [16]	VO_2_	<75.0%	>−6.00
Markov et al. [26]	VO_2_	-	~−9.00
This work	VO_2_	99.4%	−22.16

## Data Availability

The data are available upon reasonable request from the corresponding author.
